# Pancreatitis in the Setting of Vaso-occlusive Sickle Cell Crisis: A Rare Encounter

**DOI:** 10.7759/cureus.1193

**Published:** 2017-04-25

**Authors:** Badar Hasan, Talal Asif, Cody Braun, Waled Bahaj, Eslam Dosokey, Rebecca R Pauly

**Affiliations:** 1 Department of Internal Medicine, University of Missouri Kansas City (UMKC)

**Keywords:** pancreatitis, vasooclusive crisis, sickle cell

## Abstract

Acute pancreatitis is a common cause of acute abdominal pain. Gallstones and alcohol abuse account for the majority of the cases. Pancreatic ischemia is an uncommon but established cause of pancreatitis associated with connective tissue diseases, vasculitis, and shock. Our case highlights a rare case of vaso-occlusive crisis (VOC) in a patient with sickle cell (SC) disease leading to pancreatitis. Treatment remains largely conservative but exchange transfusion may be the therapy of choice in severely hypoxic patients or in patients with high pre-treatment hemoglobin S levels.

## Introduction

Acute pancreatitis is a common cause of acute abdominal pain. The incidence ranges between 13 to 45 in 100,000 among general population. Gallstones and alcohol abuse account for the majority of the cases [1]. Pancreatic ischemia is an uncommon cause of pancreatitis associated with connective tissue diseases, vasculitis, and shock [2]. Sickle cell disease (SCD) is an autosomal recessive disease with production of hemoglobin S (HbS) due to a point mutation in the beta globin gene. This causes distortion of red blood cells (RBCs) when the oxygen saturation is lowered such as during stress, infection or dehydration. The deformed RBCs cause vaso-occlusion, tissue ischemia, and infarction. We present a rare case of vaso-occlusive crisis (VOC) in a patient with SCD leading to acute ischemic pancreatitis. Our case helps to raise awareness and education on the importance of considering this critical cause of acute pancreatitis in patients with SCD. Timely diagnosis is imperative owing to a different treatment approach in this patient population.

## Case presentation

A 37-year-old African American female patient with a past medical history of homozygous sickle cell disease (HbSS) requiring multiple past hospitalizations for vaso-occlusive crises, presented to the outpatient sickle cell clinic with diffuse abdominal pain for one day. She described the pain as sharp, more severe in the epigastrium, 9/10 on a pain severity scale, worse with movement and not relieved by her oral pain medications. In the sickle cell clinic, nursing staff reported that her systolic blood pressure was 60 mmHg, diastolic blood pressure was not obtainable and she had a feeble pulse. The patient was urgently transferred to the emergency room (ER).

In the ER, her blood pressure was 78/60 mmHg, pulse 120/minute, respiratory rate 24/minute, oxygen saturation 98%, and temperature 97.8°F. On physical examination, she had a toxic appearance with scleral icterus. The patient had diffuse abdominal tenderness but no distension. There was no guarding, rigidity or rebound tenderness. Bowel sounds were audible. Cardiovascular, respiratory, and neurological examinations were unremarkable.

Immediate intravenous (IV) access was obtained. Fluid resuscitation was initiated. She required vasopressor support with norepinephrine. All baseline labs, blood and urine cultures were obtained. Broad spectrum antibiotics (vancomycin, piperacillin/tazobactam and levofloxacin) were initiated due to the undifferentiated nature of the shock at that time.

An initial complete blood count revealed hemoglobin (Hb) of 9.1 g/dl, which was below her baseline of 10 g/dl, and leukocytosis of 19.1 X 10^3^ per cubic millimeter with no left shift. The retic count was increased to 7.1% from a baseline of three percent and the hemoglobin S level was 60.6% (it should be less than 30%) [3]. The basic metabolic panel showed acute kidney injury with creatinine of 3.74 mg/dl, high anion gap metabolic acidosis with bicarbonate of 11 mmol/L, lactic acid level of 5.0 mmol/L and an anion gap of 31. Lactic acid dehydrogenase was elevated at 1539 units/L. Arterial blood gas analysis revealed a pH of 7.19. Liver function tests showed total bilirubin of 9.6 mg/dl with indirect bilirubin of 7.7 mg/dl. Serum lipase was 1511 units/L, alkaline phosphatase 149 units/L, alanine aminotransferase 88 unit/L and aspartate aminotransferase 88 units/L. These lab parameters suggested hemolysis and progression towards multi-organ failure.

**Figure 1:**
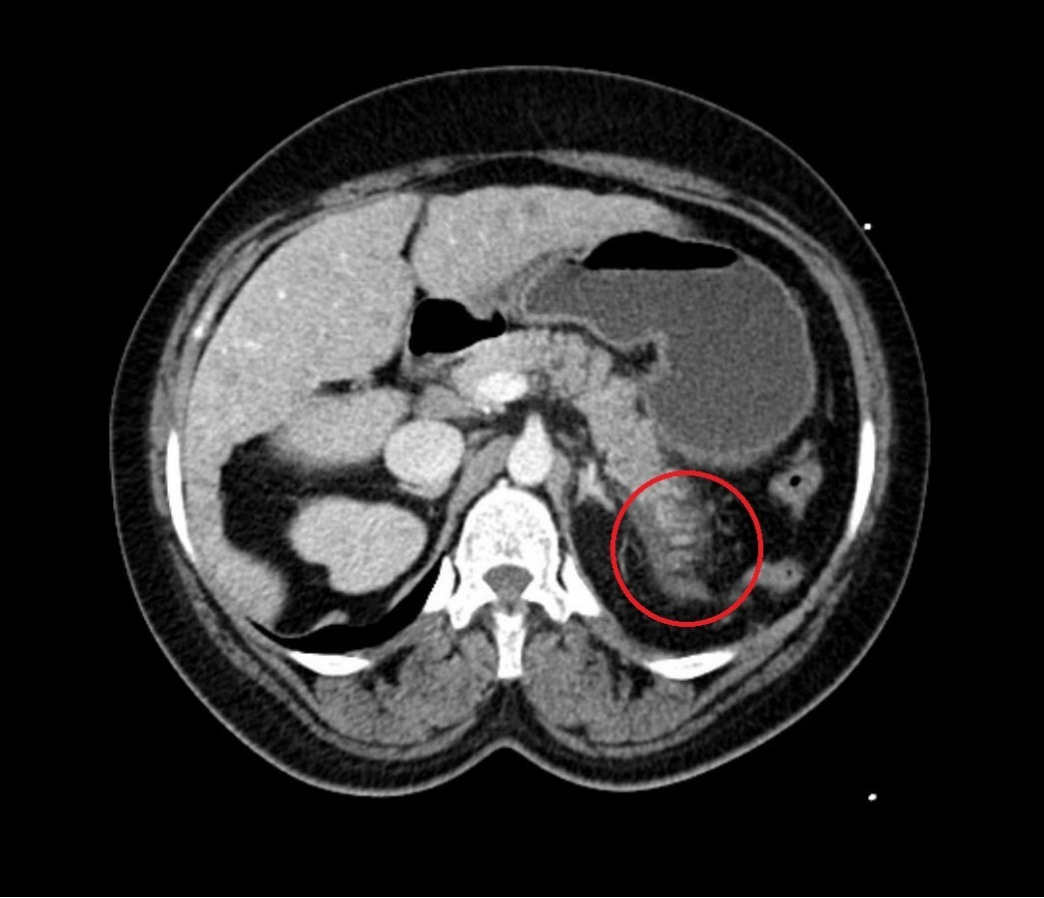
Computed tomography (CT) of abdomen showing fat stranding surrounding the pancreatic tail (red circle) consistent with acute pancreatitis.

Within two hours of presentation an ultrasound (US) and a computed tomographic (CT) scan of the abdomen were obtained. The US abdomen revealed cholelithiasis but no choledocholithiasis with normal bile duct caliber. The CT of the abdomen indicated fat stranding surrounding the pancreatic tail consistent with acute pancreatitis without necrosis or fluid collection (Figure 1).

**Figure 2:**
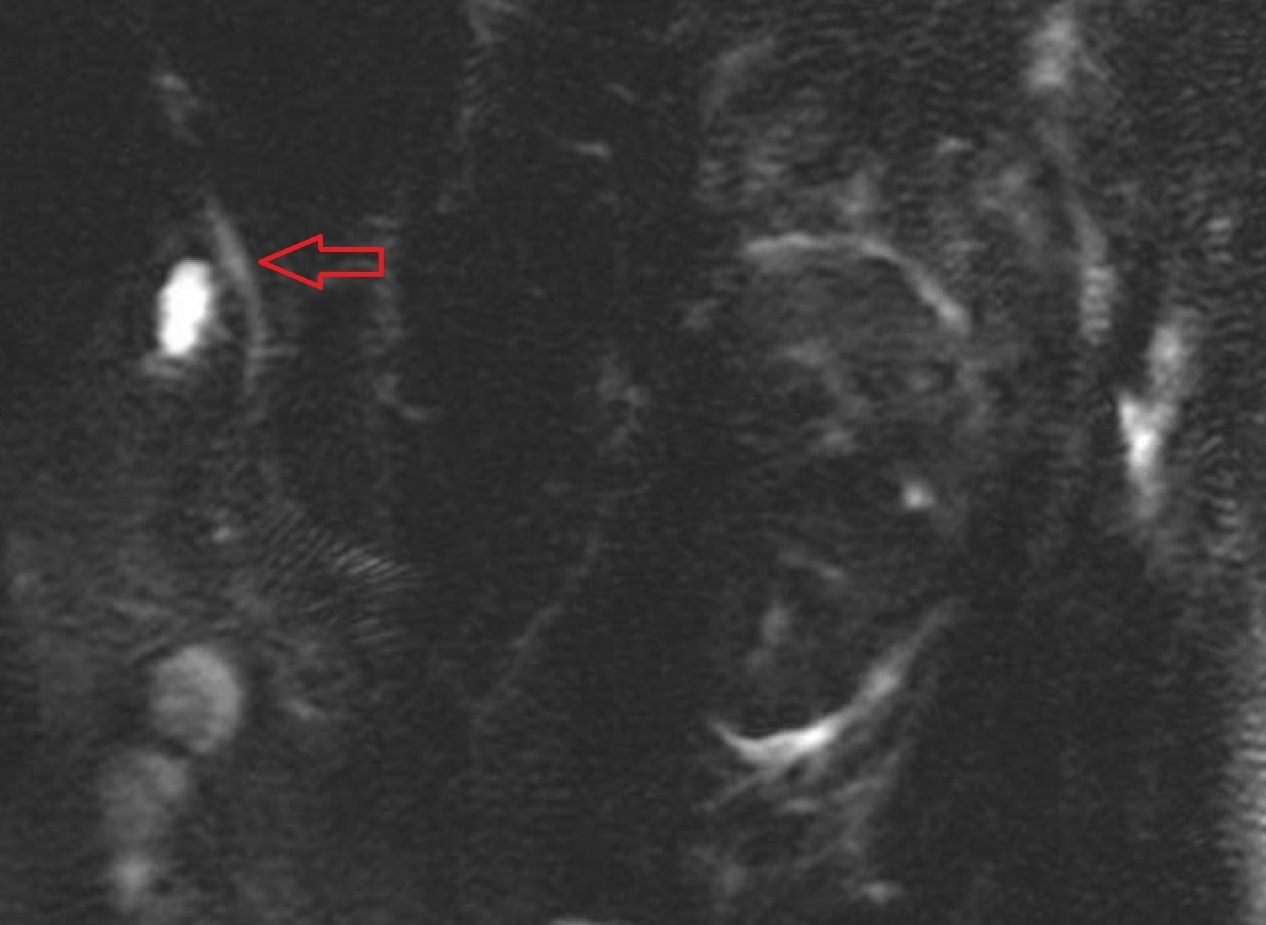
Magnetic resonance cholangiopancreatography (MRCP) showing normal caliber patent common bile duct (red arrow) with no filling defect.

The patient was transferred to the intensive care unit (ICU). Gastroenterology (GI) and hematology consultations were urgently requested. The GI team recommended treatment on the lines of septic shock secondary to ascending cholangitis. Hematology recommended immediate packed red blood cell (PRBC) exchange transfusion in the context of acute sickle cell vaso-occlusive crisis and multi-organ failure. Overnight, the patient received seven units of PRBCs exchange transfusion. The next morning, with treatment, her blood pressure and pain had improved and she was able to come off vasopressor support. The hemoglobin S level improved to 18.4%, leukocytosis resolved, the total bilirubin level came down to 5.9 mg/dl and serum creatinine declined to 2.41 mg/dl. A magnetic resonance cholangiopancreatography (MRCP) (Figure 2) was performed and it showed a common bile duct measuring 0.6 cm in maximal diameter, no intrahepatic biliary ductal dilatation, and no pancreatic ductal dilation.

The patient did not require any further exchange transfusion and continued to improve with conservative treatment.

## Discussion

Abdominal pain is a common complaint during vaso-occlusive crisis (VOC) in patients with sickle cell disease (SCD). Virtually all abdominal organs can be affected by SCD secondary to capillary engorgement, sickling, hypercoagulability and stasis in the vasa vasorum of larger vessels [4].

An estimated 10% of patients with SCD are hospitalized every year with acute abdominal pain [2]. The most frequent causes are acute cholecystitis, opioid-induced constipation, renal papillary necrosis, hepatic sequestration, splenic sequestration, urinary tract infection, peptic ulcer disease, and ischemic bowel [5]. Acute ischemic pancreatitis is a very rare complication of VOC.

The clinical features are indistinguishable from other causes of acute abdomen and represent a diagnostic challenge. Diagnosis is established on the basis of clinical suspicion, biochemical evidence, and radiological findings. In addition to abdominal pain, our patient had elevated serum lipase and computed tomographic (CT) findings of acute pancreatitis. There was no evidence of drug, alcohol, trauma, toxin, or an obstructive etiology thereby suggesting that pancreatitis was likely due to ischemic etiology from sickling [5].

Similar to any other case of acute pancreatitis, treatment is predominantly conservative including intravenous hydration, pain control and electrolyte replacement. Exchange transfusion may be the therapy of choice in patients with acute multi-organ failure. It works by diluting hemoglobin S and consequently reducing intravascular sickling, as demonstrated in our case [5]. There is no consensus on hemoglobin S goal but most authors recommend less than 20% to 30% in individuals with homozygous sickle mutation [3].

## Conclusions

Our extensive literature research found only six cases of vaso-occlusive crisis leading to acute pancreatitis. This diagnosis should be considered in any sickle cell disease patient presenting with abdominal pain since the treatment approach will be different with the possible use of exchange transfusion. Being vigilant will constitute a crucial first step, and our case helps to raise awareness and attention.
